# Prevalence of twenty-four hour urine testing in Veterans with urinary stone disease

**DOI:** 10.1371/journal.pone.0220768

**Published:** 2019-08-08

**Authors:** Calyani Ganesan, I-Chun Thomas, Shen Song, Andrew J. Sun, Ericka M. Sohlberg, Manjula Kurella Tamura, Glenn M. Chertow, Joseph C. Liao, Simon Conti, Christopher S. Elliott, John T. Leppert, Alan C. Pao

**Affiliations:** 1 Department of Medicine, Stanford University School of Medicine, Stanford, California, United States of America; 2 Department of Urology, Veterans Affairs Palo Alto Health Care System, Palo Alto, California, United States of America; 3 Geriatric Research and Education Clinical Center, Veterans Affairs Palo Alto Health Care System, Palo Alto, California, United States of America; 4 Department of Urology, Stanford University School of Medicine, Stanford, California, United States of America; 5 Department of Health Research and Policy, Stanford University School of Medicine, Stanford, California, United States of America; 6 Division of Urology, Santa Clara Valley Medical Center, San Jose, California, United States of America; 7 Department of Medicine, Veterans Affairs Palo Alto Health Care System, Palo Alto, California, United States of America; University of Oxford, UNITED KINGDOM

## Abstract

**Objective:**

The American Urological Association guidelines recommend 24-hour urine testing in patients with urinary stone disease to decrease the risk of stone recurrence; however, national practice patterns for 24-hour urine testing are not well characterized. Our objective is to determine the prevalence of 24-hour urine testing in patients with urinary stone disease in the Veterans Health Administration and examine patient-specific and facility-level factors associated with 24-hour urine testing. Identifying variations in clinical practice can inform future quality improvement efforts in the management of urinary stone disease in integrated healthcare systems.

**Materials and methods:**

We accessed national Veterans Health Administration data through the Corporate Data Warehouse (CDW), hosted by the Veterans Affairs Informatics and Computing Infrastructure (VINCI), to identify patients with urinary stone disease. We defined stone formers as Veterans with one inpatient ICD-9 code for kidney or ureteral stones, two or more outpatient ICD-9 codes for kidney or ureteral stones, or one or more CPT codes for kidney or ureteral stone procedures from 2007 through 2013. We defined a 24-hour urine test as a 24-hour collection for calcium, oxalate, citrate or sulfate. We used multivariable regression to assess demographic, geographic, and selected clinical factors associated with 24-hour urine testing.

**Results:**

We identified 130,489 Veterans with urinary stone disease; 19,288 (14.8%) underwent 24-hour urine testing. Patients who completed 24-hour urine testing were younger, had fewer comorbidities, and were more likely to be White. Utilization of 24-hour urine testing varied widely by geography and facility, the latter ranging from 1 to 40%.

**Conclusions:**

Fewer than one in six patients with urinary stone disease complete 24-hour urine testing in the Veterans Health Administration. In addition, utilization of 24-hour urine testing varies widely by facility identifying a target area for improvement in the care of patients with urinary stone disease. Future efforts to increase utilization of 24-hour urine testing and improve clinician awareness of targeted approaches to stone prevention may be warranted to reduce the morbidity and cost of urinary stone disease.

## Introduction

Twenty-four-hour urine testing is employed in the metabolic evaluation and treatment of patients with urinary stone disease (USD). Clinicians use the 24-hour urine collection, along with chemical analysis of kidney stone composition, to diagnose urinary or systemic abnormalities responsible for kidney stone formation. The 24-hour urine collection can also be used to guide dietary and pharmacologic treatments for USD. Currently, the American Urological Association (AUA) guidelines recommend 24-hour urine testing for evaluating and treating recurrent stone formers and high-risk or interested first time stone formers [1].

While there is sound physiological rationale for incorporating 24-hour urine testing in assessing USD risk, variability in the application of, and effectiveness of strategies incorporating, 24-hour urine testing are not well characterized [2]. To address these gaps in evidence, we used a large national cohort of patients in the Veterans Health Administration (VHA) for the following objectives: 1) estimate the prevalence of 24-hour urine testing in Veterans with USD across the United States; 2) identify demographic, geographic, and clinical characteristics of patients with USD who are more or less likely to receive 24-hour urine testing; and 3) characterize healthcare utilization associated with 24-hour urine testing. We hypothesized that the prevalence of 24-hour urine testing remains low despite clinical practice guidelines recommending metabolic evaluation of patients with USD, and that application of 24-hour urine testing varies widely by patient-specific and facility-level characteristics.

## Materials and methods

### Data and study population

**Fig 1:**
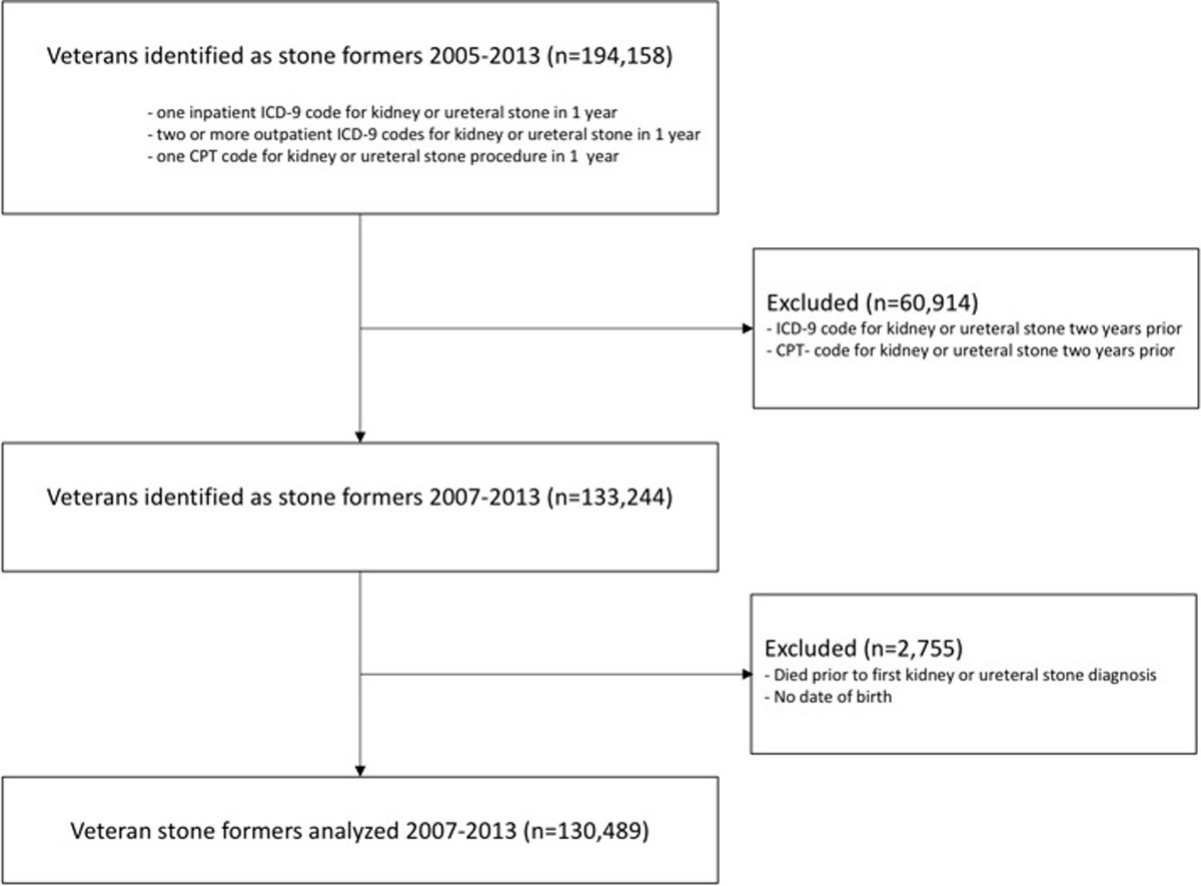
Flow chart showing patient cohort selection and exclusion.

The Stanford Institutional Review Board and Veterans Administration Research and Development Committee (FWA00000929) approved this study and waived informed consent under the following protocol, IRB 6 Registration #4947. All records were fully anonymized before accessed. We accessed national VHA data stored in the Corporate Data Warehouse (CDW), hosted by the Veterans Affairs Informatics and Computing Infrastructure (VINCI), to identify patients with USD from 2007 through 2013. We defined “stone formers” as patients who had one or more inpatient ICD-9 codes for kidney or ureteral stones, two or more outpatient ICD-9 codes for kidney or ureteral stones, or one or more CPT codes for kidney or ureteral stone procedures in a single year [3]. We limited our cohort to patients who were free from a kidney or ureteral stone diagnosis or procedure in the two years before entry into the cohort to ascertain how clinicians respond to a new stone event episode (Fig 1). Each patient entered the cohort once, at the time of his or her first qualification as a stone former during the observation period.

### Primary outcome

We identified the fraction of stone formers who completed a 24-hour urine collection from time of entry into the observation period through 2014. We used Logical Observation Identifiers Names and Codes (LOINC) to define a 24-hour urine collection as the presence of a 24-hour urine measurement for calcium, oxalate, citrate or sulfate. We based our definition on the assumptions that 24-hour testing for urine calcium is a sensitive proxy for USD evaluation and that 24-hour urine testing for oxalate, citrate, or sulfate is a specific proxy. We chose not to define a 24-hour urine collection as requiring the presence of all parameters that are included in a 24-hour urine stone risk panel due to our concern that it would lead to under-ascertainment of 24-hour urine testing. To ascertain if VHA includes data from 24-hour urine collections processed by an outside laboratory, we queried 24-hour urine data from our local VHA facility, which sends out 24-hour urine samples to Quest Diagnostics for measurement of urine calcium, oxalate, citrate, and sulfate, and we confirmed that these test results were recorded in the VHA.

### Patient characteristics

We compared demographic and clinical characteristics among stone formers who completed a 24-hour urine collection and those who did not. We examined the following factors we considered to be associated with stone risk: age, sex, race/ethnicity, history of cardiovascular disease (CVD), diabetes, cancer, hyperparathyroidism, paralysis, chronic obstructive pulmonary disease (COPD), dementia, prior 24-hour urine collection, and geographic region (broadly characterized as Northeast, Southeast, West, Midwest). To assess if a stone former received specialty stone care, we identified provider codes associated with a nephrology or urology clinic visit 6 months after their initial stone diagnosis (Table 1).

**Table 1:** Nephrology and urology clinic visit codes.

	Provider Codes	Clinic Stop Codes
**Nephrology**	070929, 151003,11512,181018,182515,040403, 150216	313
**Urology**	070951, 118200, 118000, 183400	414

### Statistical analysis

We first categorized stone formers into those who had a 24-hour urine collection and those who did not. We compared continuous variables using Student’s t-tests, and we compared categorical variables with the *x*^2^ test. We used multivariable logistic regression to estimate the odds ratios (ORs) and 95% confidence intervals (95% CI) of having a 24-hour urine collection after adjusting for age, sex, race/ethnicity, geography, prior kidney stone procedure, prior 24-hour urine collection, study entry criteria (outpatient, inpatient, or kidney stone procedure), and Charlson comorbidity index. We used the location associated with stone diagnosis code to assign each patient to one of 150 facilities across the VHA. We then compared the use of 24-hour urine testing across VHA facilities. We considered 2-tailed p-values <0.05 as statistically significant. We conducted our analysis using SAS version 9.4 (Cary, NC, USA).

## Results

We identified 130,489 patients with USD from 2007 through 2013 and found that 19,288 of these patients (14.8%) completed at least one 24-hour urine collection (Table 2). In both groups, male sex (92.7% and 94.9%) and White race (79.5% and 77.4%) were most common. Stone formers who completed a 24-hour urine collection were more likely to qualify for the cohort through two or more outpatient encounters (70.5% versus 61.1%, p<0.001), whereas those who did not complete a 24-hour urine collection were more likely to qualify for the cohort through an inpatient encounter (12.9% versus 7.9%, p<0.001) or stone procedure (26% versus 21.5%, p<0.001). Stone formers who completed 24-hour urine testing received more stone procedures compared to those who did not, with the median number of procedures being 1 (IQR 0–2) versus 0 (IQR, 0–1), respectively (p<0.001). Sixty-four percent of stone formers visited either a nephrologist or urologist 6 months after their initial stone diagnosis, and 18.7% of these stone formers completed a 24-hour urine collection (Table 3).

**Table 2:** Baseline characteristics of stone formers with and without a 24-hour urine collection.

Characteristics	With 24-hour	Without 24-hour	p-value
	(n = 19,288)	(n = 111,201)	
Age (mean ± SD, years)	58.3 ± 12.5	61.2 ± 13.8	<0.001
Sex (n,%)			
Male	17,884 (92.7)	105,541 (94.9)	<0.001
Female	1404 (7.3)	5660 (5.1)	<0.001
Race/Ethnicity			
White	15,331 (79.5)	86,013 (77.4)	<0.001
Black	1760 (9.1)	12,301 (11.1)	<0.001
Other or unknown	1864 (9.7)	9900 (8.9)	<0.001
Stone Diagnosis			
Inpatient	1525 (7.9)	14,326 (12.9)	<0.001
Outpatient	13,607 (70.5)	67,931 (61.1)	<0.001
Procedure	4156 (21.5)	28,944 (26)	<0.001
Stone procedures (median, IQR)	1 (0–2)	0 (0–1)	<0.001
Charlson Comorbidity Index (mean ± SD)	2.1 ± 2.3	2.5 ± 2.6	
Individual Conditions (n,%)			
CVD^a^	6405 (33.2)	47,577 (42.8)	<0.001
Diabetes Mellitus	6042 (31.3)	35,528 (31.9)	0.08
Cancer	2580 (13.4)	21,527 (19.1)	<0.001
Paralysis	574 (3.0)	3527 (3.2)	0.15
COPD	4617 (23.9)	29,578 (26.6)	<0.001
Dementia	365 (1.9)	4202 (3.8)	<0.001
Hyperparathyroidism	493 (2.6)	324 (0.3)	<0.001

^a^CVD: includes cardiovascular disease, myocardial infarction, heart failure and peripheral vascular disease

**Table 3:** Stone specialty (Nephrology or Urology) provider care 6 months after stone diagnosis.

Stone former (n,%)	Nephrology or Urology Visit (n = 84,086)	No Specialty Visit (n = 46,393)	p-value
With 24-hour	15,722 (18.7)	3556 (7.6)	<0.001
Without 24-hour	68,364 (81.3)	42,837 (92.4)	<0.001

We assessed differences in comorbid illnesses between the two groups (Table 2). Stone formers who completed a 24-hour urine collection had fewer comorbid illnesses compared to those who did not (Charlson Comorbidity Index of 2.1 versus 2.5). In addition, specific diagnoses associated with USD were strongly associated with completion of a 24-hour urine collection. For example, patients with a history of hyperparathyroidism had 8.6-fold higher odds of completing a 24-hour urine collection compared to those who did not.

Using multivariable regression, we identified patient-specific factors associated with 24-hour urine testing in stone formers (Table 4). Stone formers who completed a 24-hour urine collection were more likely to be young or of White race. The odds of 24-hour urine testing were also directly related to the number of stone procedures (ORs are calculated in reference to a stone former without a stone procedure): 1–2 procedures (OR 1.91, 95% CI 1.84 to 1.99), 3–4 procedures (OR 2.89, 95% CI 2.72 to 3.06), or 5–6 procedures (OR 3.47, 95% CI 3.15 to 3.81). A stone former was much more likely to complete a 24-hour urine test if he or she completed a 24-hour urine test prior to the observation period (OR 3.06, 95% CI 2.80 to 3.34).

**Table 4:** Multivariable logistic regression reporting the odds of completing a 24-hour urine collection.

Variable	Odds Ratio	Confidence Interval	p-value
Age (per decade)	0.85	0.83–0.86	<0.001
Female vs. Male	1.07	1.00–1.15	.056
White vs. Black	1.28	1.20–1.36	<0.001
Stone Procedure			
0	Ref		
1–2	1.91	1.84–1.99	<0.001
3–4	2.89	2.72–3.06	<0.001
5–6	3.47	3.15–3.81	<0.001
Charlson Comorbidity Index			
0	Ref		
1	0.89	0.85–0.94	<0.001
2	0.86	0.81–0.90	<0.001
3	0.86	0.81–0.92	<0.001
4	0.78	0.73–0.84	<0.001
> = 5	0.63	0.59–0.68	<0.001
Prior 24-hour urine test	3.06	2.80–3.34	<0.001
Region			
Midwest	Ref		
West	1.20	1.15–1.26	<0.001
Northeast	0.71	0.67–0.75	<0.001
Southwest	0.68	0.65–0.72	<0.001

**Fig 2:**
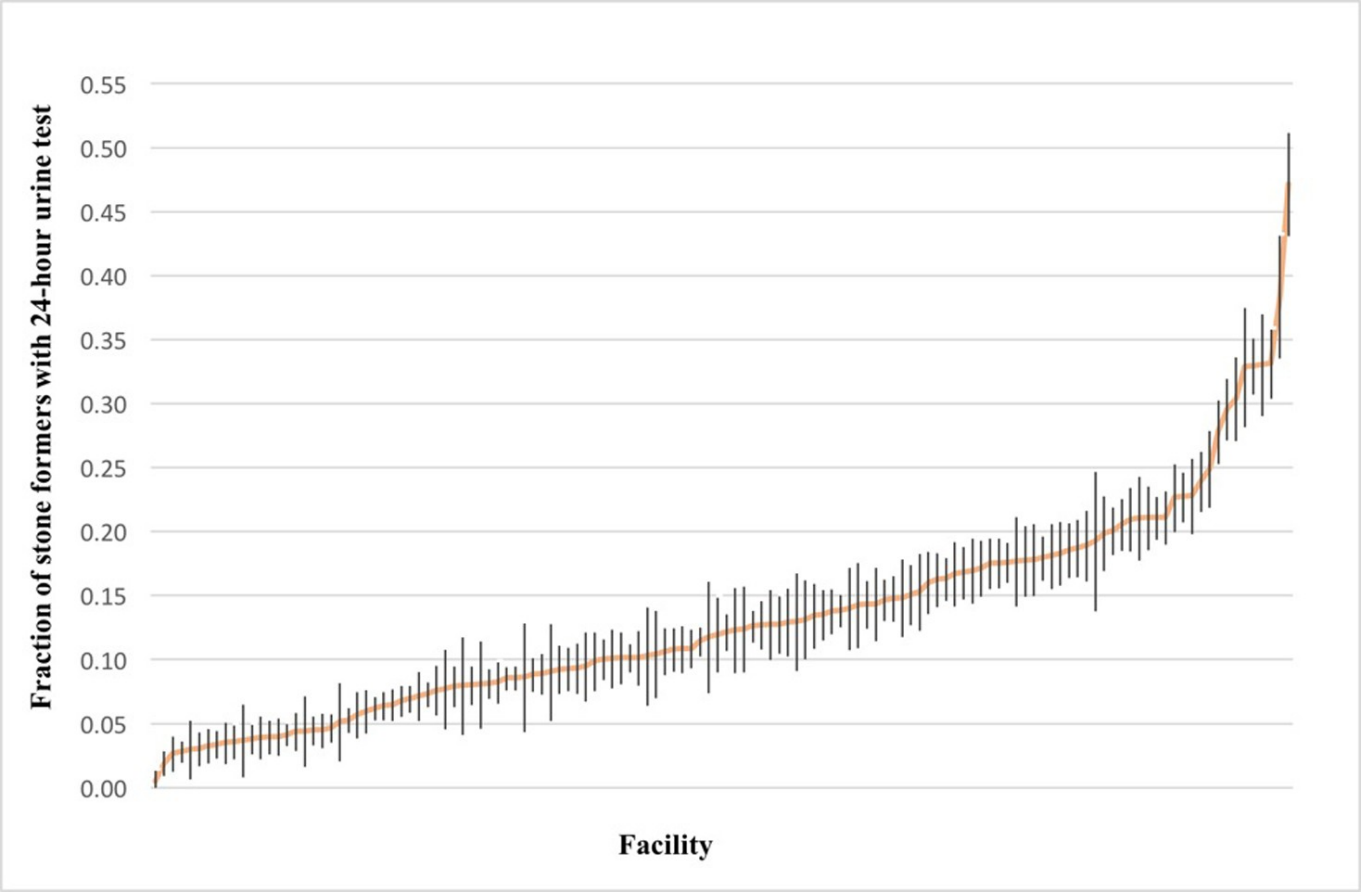
Prevalence of 24-hour urine testing by VHA facility. Each bar reflects the standard error around the fraction estimate for each VHA facility.

Next we examined geographic and facility-level variation in 24-hour urine testing in stone formers across the country (Table 4). Relative to stone formers in the Midwest, patients treated in the West were more likely (OR 1.20, 95% CI 1.15 to 1.26) and patients treated in the Northeast (OR 0.71, 95% CI 0.67 to 0.75) and Southeast (OR 0.68, 95% CI 0.65 to 0.72) were less likely to be tested. To further explore this geographic variation, we estimated the prevalence of 24-hour urine testing in stone formers at each of the VHA facilities. Among the 150 VHA facilities delivering care to stone formers, the unadjusted prevalence of 24-hour urine testing ranged between 1 and 40% (Fig 2). We also compared demographic and clinical characteristics of patients seen in the top and bottom decile of VHA facilities that administer 24-hour urine testing and found them to be similar (Table 5). While the volume of patients with USD was modestly higher in the top decile compared to the bottom decile of VHA facilities (9835 versus 7751 patients, respectively), the average number of stone procedures for each stone former within the observation period was similar (0.8 ± 1.4 versus 0.7 ± 1.3).

**Table 5:** Baseline characteristics of stone formers with and without a 24-hour urine collection in the top and bottom decile of VHA facilities that administer 24-hour urine testing.

Characteristics		Top Decile			Bottom Decile		p-value^a^
	Total	With 24-hour	Without 24-hour	Total	With 24-hour	Without 24-hour	
	(n = 9835)	(n = 3052)	(n = 6783)	(n = 7751)	(n = 241)	(n = 7510)	
Age (mean ± SD)	60.6 ± 13.5	58.6 ± 12	61.5 ± 14.1	60.3 ± 13.6	55.5 ± 13.1	60.4 ± 13.6	0.79
Sex (n,%)							
Male	9268 (94.2)	2853 (93.5)	6415 (94.6)	7329 (94.6)	217 (90)	7112 (94.7)	0.36
Female	567 (5.8)	199 (6.5)	368 (5.4)	422 (5.4)	24 (10)	398 (5.3)	
Race (n,%)							
White	7437 (75.6)	2395 (78.5)	5042 (74.3)	5415 (69.9)	178 (73.9)	5237 (69.7)	<0.001
Black	796 (8.1)	188 (6.2)	608 (9)	934 (12.1)	28 (11.6)	906 (12.1)	
Other/Unknown	1601 (16.3)	469 (15.4)	1133 (16.7)	1402 (18)	35 (14.5)	1367 (18.2)	
Stone Diagnosis (n,%)							
Inpatient	1067 (10.8)	242 (7.9)	825 (12.2)	844 (10.9)	18 (7.5)	826 (11)	<0.001
Outpatient	6740 (68.5)	2309 (75.7)	4431 (65.3)	5114 (66)	181 (75.1)	4933 (65.7)	
Procedure	2028 (20.6)	501(16.4)	1527 (22.5)	1793 (23.1)	42 (17.4)	1751 (23.3)	
Number of procedures (mean ± SD)	0.8 ± 1.4	1.2 ± 1.7	0.6 ± 1.2	0.7 ± 1.3	1.5 ± 2.1	0.7 ± 1.2	<0.001
Charlson Comorbidity Index (mean ± SD)	2.3 ± 2.5	1.9 ± 2.1	2.4 ± 2.6	2.3 ± 2.5	1.9 ± 2.2	2.3 ± 2.6	0.13
Region (n,%)							
Midwest	1219 (12.4)	292 (9.6)	927 (13.7)	1270 (16.4)	31 (12.9)	1239 (16.5)	<0.001
Northeast	1285 (13.1)	363 (11.89)	922 (13.6)	1405 (18.1)	54 (22.4)	1351(18)	
Southeast	2870 (29.2)	969 (31.8)	1901 (28)	3560 (45.9)	108 (44.8)	3452(46)	
West	4461 (45.4)	1428 (46.8)	3033 (44.7)	1516 (19.6)	48 (19.9)	1468 (19.6)	

^a^p-value compares characteristics between the total population in the bottom and top decile of facilities

## Discussion

In this study, we found that fewer than one in six patients with USD completed 24-hour urine testing despite guidelines recommending its use for secondary prevention. The low prevalence of 24-hour urine testing in stone formers demonstrates that clinicians are not routinely incorporating this diagnostic tool and may be falling short of adopting targeted approaches to stone prevention. We identified patient-specific and facility-level factors associated with 24-hour urine testing to determine whether one set of factors dominated. Stone formers who completed a 24-hour urine collection were more likely to do so through an outpatient encounter and were healthier, with lower rates of cardiovascular disease, dementia, and cancer, suggesting that patients who are burdened by co-morbid illness are less likely to collect a 24-hour urine sample. Stone formers were also more likely to complete a 24-hour urine test if they had more than one stone procedure or if they had completed a 24-hour urine test prior to the observation period. These findings suggest that clinicians select patients for 24-hour urine testing who have an increased risk of stone recurrence; moreover, once patients are placed on a “metabolic care pathway” guided by 24-hour urine testing, they are more likely to undergo future testing.

We also found striking facility-level variation in 24-hour urine testing. Among the 150 VHA facilities delivering care to Veterans, the unadjusted proportion of stone formers receiving 24-hour urine testing ranged between 1 and 40%. This wide variation in 24-hour urine testing in stone formers across VHA facilities cannot be explained solely by differences in patient-level characteristics. For example, the average number of stone procedures performed for each stone former in the top and bottom decile of VHA facilities ordering 24-hour urine collections was nearly equal, indicating that stone formers in the top decile are not different from those in the bottom decile with respect to their need for USD procedures. Notably, even the top decile of VHA facilities ordering 24-hour urine collections do so at a rate that falls under 40% of patients with USD, highlighting a divide between recommended and real-world practice of incorporating 24-hour urine testing as a strategy for USD prevention.

To date, few studies have examined the role of 24-hour urine testing in secondary prevention of USD [4–7]. Milose et al. estimated the frequency of 24-hour urine testing to be between 7 and 7.9% in high risk stone formers [7]. Alruwaily et al. used Litholink Corporation data to assess follow-up 24-hour urine testing (i.e., a second 24-hour urine collection) and found the mean rate to be between 11 and 12% [4]. Our study examined a large national cohort of stone formers with varying stone risk profiles and found similarly low rates of testing. It is important to emphasize that our study may have included low risk or first time stone formers in our analytic cohort, which opens up the possibility that 24-hour urine testing may not have been indicated in some stone formers. Future studies are necessary to determine the reasons underlying the low observed rates and regional differences in 24-hour urine testing in stone formers.

Prior studies examining the relationship between 24-hour urine testing and specialty stone care have shown conflicting results. On the one hand, Milose et al. showed that the odds of receiving a 24-hour urine collection increases 2.9-fold when a stone former is seen by a nephrologist and increases more than 3-fold when a stone former is seen by a urologist [7]. On the other hand, Dauw et al. showed that the odds of receiving a repeat 24-hour urine collection decreases 0.76-fold when a stone former is seen by a urologist but increases 1.32-fold when a stone former is seen by a nephrologist [5]. In our study, we found that while 64% of stone formers visited a urologist or nephrologist, only 18.7% of these stone formers completed a 24-hour urine collection, suggesting that the low prevalence of 24-hour urine testing is more likely a function of individual provider preference or regional/facility-level differences rather than access to specialty stone care. These findings highlight the need for stone specialists, who typically care for high risk stone formers, to incorporate 24-hour urine testing in secondary prevention of USD.

This study has several strengths. First, the VHA is a relevant population for the study of USD. Veteran patients tend to be older than civilian patients and have higher rates of co-morbid illnesses that place them at particular risk for USD [8]. Second, we incorporated data from an integrated health system with both inpatient and outpatient claims. The patient sample was large and diverse in terms of age, race, geography and the presence or absence of multiple comorbid conditions. Third, the VHA may be an ideal setting for evaluating determinants of 24-hour urine testing because clinicians in the VHA do not require prior authorization from insurance companies to order 24-hour urine collections, which could reduce detection bias that may be present in civilian health care systems.

This study also has several limitations. First, our study population was comprised of Veterans, the majority of whom are men, which limits generalizability to women or persons unaffiliated with military service. Second, we defined a 24-hour urine collection to be a 24-hour urine measurement of calcium, oxalate, citrate, or sulfate, which is a subset of the measurements found in a typical 24-hour urine test. We included calcium which we believed to be a highly sensitive parameter as well as oxalate, citrate and sulfate which we believed to be a highly specific parameter to diminish false positives and negatives. We also limited our analysis to examining the rate of patients completing a 24-hour urine test rather than the rate of clinicians *ordering* a 24-hour urine test. It is possible that a discrepancy between these two rates exists in the VHA and could indicate patient or facility factors that impede successful completion of 24-hour urine testing. Currently the CDW dataset does not organize order names for 24-hour urine tests in a standardized format as it does for completed 24-hour tests, which are each associated with a specific set of LOINC codes. A lack of standardization in order names for 24-hour urine tests makes it difficult to know if order names used to query the CDW dataset will encompass all order names that are used for 24-hour urine tests across the 150 VHA facilities. Future studies are thus needed to estimate the rate of 24-hour urine test ordering in the VHA (or elsewhere) and to determine if this rate differs from the rate of 24-hour urine test completion. Third, we used ICD-9 codes to identify urinary stone disease diagnoses, which opens the possibility that a diagnosis could be either over-coded (e.g., emergency room visit for abdominal pain may be coded as a kidney stone) or under-coded (e.g. outpatient visit for multiple comorbid illnesses may not include a kidney stone code). Fourth, we could not specifically exclude stone formers for whom 24-hour urine testing was not indicated (e.g. un-interested first time or low risk stone formers). Fifth, it is possible that we may not have captured data from all VHA facilities that send 24-hour urine samples to an outside laboratory for processing. To address this concern, we queried 24-hour urine data from our local VHA facility, which sends out 24-hour urine samples to an outside laboratory for measurement of urine calcium, oxalate, citrate, and sulfate. We confirmed that these 24-hour urine test results were recorded in the VHA, but we acknowledge that we do not know if this would be the case for all VHA facilities. Finally, we did not capture medical care for stone formers who receive care outside the VHA.

## Conclusions

In conclusion, the 24-hour urine collection is acknowledged to be a valuable diagnostic tool for clinicians aiming to identify and treat metabolic risk in stone formers, yet fewer than one in six patients with USD undergo testing. This low testing frequency does not appear to be due to lack of access to specialty care because the majority of stone formers visited either a urologist or nephrologist after a stone diagnosis. Moreover, 24-hour urine testing in stone formers varied widely by geographic region and VHA facility. These regional and facility-level differences in utilization of 24-hr urine testing appear to reflect fixed practice-patterns with respect to how clinicians approach USD prevention. Future efforts to increase utilization of 24-hour urine testing and improve clinician awareness of targeted approaches to stone prevention may be warranted to reduce the morbidity and cost of USD.
